# Ripening-Induced Changes in the Nutraceutical Compounds of Differently Coloured Pepper (*Capsicum annuum* L.) Breeding Lines

**DOI:** 10.3390/antiox11040637

**Published:** 2022-03-26

**Authors:** Zsófia Kovács, Janka Bedő, Bánk Pápai, Andrea Kitti Tóth-Lencsés, Gábor Csilléry, Antal Szőke, Éva Bányai-Stefanovits, Erzsébet Kiss, Anikó Veres

**Affiliations:** 1 Institute of Genetics and Biotechnology, Hungarian University of Agriculture and Life Sciences, 2100 Gödöllő, Hungary; bedo.janka@uni-mate.hu (J.B.); bankitooo@gmail.com (B.P.); toth-lencses.andrea.kitti@uni-mate.hu (A.K.T.-L.); szoke.antal@uni-mate.hu (A.S.); kiss.erzsebet@uni-mate.hu (E.K.); veres.aniko@uni-mate.hu (A.V.); 2 PepGen Kft., 1114 Budapest, Hungary; csillerygabor48@gmail.com; 3 Institute of Food Science and Technology, Hungarian University of Agricultural Sciences, 1118 Budapest, Hungary; stefanovitsne.banyai.eva@uni-mate.hu

**Keywords:** *Capsicum*, pepper, anthocyanin, antioxidant, secondary metabolites, gene expression, R2R3-MYB

## Abstract

To date, several research studies addressed the topic of phytochemical analysis of the different coloured pepper berries during ripening, but none discussed it in the case of purple peppers. In this study we examine whether the anthocyanin accumulation of the berries in the early stages of ripening could result in a higher antioxidant capacity due to the elevated amount of polyphenolic compounds. Therefore, enzymatic and non-enzymatic antioxidant capacity was measured in four distinct phenophases of fruit maturity. Furthermore, the expression of structural and regulatory genes of the anthocyanin biosynthetic pathway was also investigated. An overall decreasing trend was observed in the polyphenolic and flavonoid content and antioxidant capacity of the samples towards biological ripeness. Significant changes both in between the genotypes and in between the phenophases were scored, with the genotype being the most affecting factor on the phytonutrients. An extreme purple pepper yielded outstanding results compared to the other genotypes, with its polyphenolic and flavonoid content as well as its antioxidant capacity being the highest in every phenophase studied. Based on our results, besides MYBa (*Ca10g11650*) two other putative MYBs participate in the regulation of the anthocyanin biosynthetic pathway.

## 1. Introduction

Pepper (*Capsicum annuum* L.) is one of the most consumed vegetable and spice crops. Despite its numerous characteristics that determine the consumer acceptance of the fruit, it is still highly sought after worldwide. While shape and pungency are also determining factors, colour in particular ranks very high in the perceived palatability and perceived flavour intensity of fruits/vegetables [1,2,3]. Colours of the immature pepper fruits vary from ivory to green, and newer varieties may even exhibit different shades of purple, whereas the colouration of mature fruits of cultivated peppers ranges from white through yellow, orange and red to even a brownish hue. The main determinants of colour in pepper fruit are the chlorophyll, carotenoid and anthocyanin pigments [4,5,6,7]. These pigments are stored in different cell compartments; chlorophylls are located within the chloroplasts, carotenoids are stored in the chromoplasts, while anthocyanins and other flavonoids are accumulated in vacuoles in the cells’ cytoplasm.

Fruit colour is of upmost importance since pigments affecting the colouration further contribute to a wide variety of functions such as protection against various abiotic stresses, e.g., UV light, excess light, cold temperature, and pathogens. More importantly, they contribute to the flavour and nutraceutical properties since most of these compounds exhibit antioxidant activity. Thus, these phytochemicals possess potential health benefits for a human diet. For instance, carotenoids are involved in the scavenging of both peroxyl radicals and singlet oxygen species (O^2−^) [8]. A carotenoid-rich diet could lower risk of several types of cancer and cardiovascular disease. Capsanthin—a red pigment which is synthesized exclusively in *Capsicum* spp.—proved to suppress hydroperoxide formation, and since they decompose slower than other carotenoids found in peppers, their radical scavenging activity lasts longer [9]. From nutraceutical aspects, various pepper fruits’ carotenoid accumulation is among the highest in plants [10]. A teaspoon of its powder provides the Recommended Dietary Allowance for vitamin A of an adult person after the conversion of the precursor carotenoids to vitamin A [11].

Flavonoids are a large class of compounds that are important antioxidants. Numerous studies indicate that a flavonoid-rich diet reduces the risk of coronary heart disease, stroke and lung/breast cancer [12]. Their antioxidant properties are determined by both the chemical structure and by the redox properties; based on these, they can serve as O^2−^ quenchers, hydrogen donors or reducing agents. Further, they are efficient against lipid oxidation [13,14,15].

Health-conscious diets are on the rise, and currently there is an ever-growing interest towards flavonoid/anthocyanin-rich functional foods, hence purple fruits and vegetables are commercially valued. The biosynthesis of flavonoids, and subsequently anthocyanins, is regulated by the MYB transcription factors. The production of anthocyanins branches off from the flavonoid pathway and they are synthesized via the early biosynthetic genes (EBGs), e.g., chalcone synthase (*CHS*), chalcone isomerase (*CHI*), flavanone-3-hydroxylase (*F3H*), and the late biosynthetic genes (LBGs) such as dihydroflavonol reductase (*DFR*), anthocyanidin synthase (*ANS*), flavonoid 3′5′-hydroxylase (*F3′5′H*), glutathion S-transferase (*GST*), and anthocyanidin 3-O-glucosyltransferase (*UFGT*) [16]. The regulation of the pathway is well studied, although there are still conflicting results which have yet to be clarified. In general, it is agreed that a regulatory complex (MBW), taking up from MYB, bHLH and WD40 transcription factors, initiates the transcription of the LBGs. In the case of pepper, three MYB coding genes are located next to each other on the 10th chromosome. Out of these, so far only the so-called MYBa is said to participate in the biosynthesis [17,18].

Many studies examined the phytochemical composition of different pepper cultivars, but there are only a few which monitor the changes of these compounds upon maturation [19]. In this study, we take differently coloured breeding lines to determine how the colouration affects the nutraceutical properties of the peppers, as well as how the phytochemical composition changes during maturation. Furthermore, we investigate the regulatory mechanism of the anthocyanin biosynthesis with emphasis being put on the MYB transcription factors and their putative role. By this, we can rule out whether the anthocyanin build-up, hence the higher amount of polyphenols in the berries, could add to the overall antioxidant capacity of the pepper. This work could not only assist breeders to select for candidate accessions with great nutritional properties as partners in breeding programs but may also shed light on the basis of the transcriptional regulation of the anthocyanin biosynthesis.

## 2. Materials and Methods

Mutant pepper breeding lines used for this study were provided by the PepGen Kft. (Budapest, Hungary). In addition, 5 anthocyanin mutant *C. annuum* peppers, a *C. annuum* ‘Soroksári’ cultivar—which does not synthetize anthocyanins—and an extreme purple *C. chinense* ‘Pimienta de Neyde’ (‘Pim. Ney.’) cultivar were applied (Table 1).

The plants were grown under greenhouse conditions. Ten fruits were gathered at 4 distinct phenophases of the ripening from each genotype. In addition to the two economically ripe stages, green stage 1 (GS1)—20 days post anthesis (dpa), green stage 2 (GS2)—30 dpa, the breaker—40 dpa, when the berries are turning into their biologically ripe colour, and the full biological ripeness stage—60 dpa, were examined. Fruits were cut, seeds, placenta, calyx and pedicel were removed, and the remaining pericarp was flash-frozen in liquid N_2_. Samples were stored at −70 °C until further use.

### 2.1. Sample Preparation

For more precise comparison of the breeding lines, during sample preparation we opted for the most measurements to be carried out from the same set of extractions. For the total monomeric anthocyanin (TMA), total phenolic content (TPC) and ferric reducing ability of plasma (FRAP) assays samples (pericarp) were crushed and homogenized in liquid N_2_. The extraction was carried out with MeOH:H_2_O:HCOOH (60:39:1 *v*/*v*%). Extracts were centrifuged at 4 °C at 4300 rpm for 20 min and the resulting supernatant was used. Until the measurements the prepared samples were stored at −32 °C. For the total flavonoid content (TFC) assay, fruit samples without seeds were sliced and extracted with Milli-Q water in a ration of 1:10. To determine the total carotenoid content (TC), samples were extracted with EtOH:acetone (1:1 *v*/*v*%), filtered and kept in dark at −32 °C until use.

Samples for the enzymatic activity of catalase (CAT) and peroxidase (POD) measurements were ground in cold sodium phosphate buffer (25 mM, pH 7.8), supplemented with 0.8 g/l PVP and 1 mM EDTA, and were centrifuged for 20 min at 12,000× *g* at 4 °C. Supernatant was applied for the studies. For the superoxide dismutase (SOD) assay, 50 mM NaPO_4_ buffer was supplemented with 1 mM EDTA and 2 *w*/*v*% PVP was applied. Samples were centrifuged for 20 min at 12,000× *g* at 4 °C and the supernatant was used for the studies. Until the measurements, samples were stored at −32 °C. For each measurement 3 technical replicates were applied.

### 2.2. Total Monomeric Anthocyanin Content (TMA)

Total monomeric anthocyanin content was measured by a pH differential method as described by Lee et al. [20]. To the samples KOH (pH 1.0) and sodium acetate (NaOAc) (pH 4.5) buffers were measured. After 15 min, absorbance was recorded by a Jenway 6105 UV/Vis spectrophotometer at λ = 520 nm and λ = 700 nm. Total monomeric anthocyanin content was calculated by the following formula:

$$\mathrm{TMA}\;(\mathrm{cyanidin}-3-\mathrm{glucosyde}\;\mathrm{mg}/L)=\frac{A\times MW\times Df\times 10^{3}}{\varepsilon \times l}$$

where A = (A_520nm_ − A_700nm_)pH 1.0 − (A_520n_ − A_700nm_)pH 4.5, MW(molecular weight) = 449.2 g/mol for cyanidin-3-glucosyde, Df = Dilution factor, ε = 26,900 molar extinction coefficient for cyanidin-3-glucosyde, l = pathlength in cm. Results are expressed as µg cyanidin-3-glucosyde/g dry weight (dw) (µg cy-3-glu/g).

### 2.3. Total Polyphenolic Content (TPC)

Total soluble polyphenols were measured with Folin–Ciocalteu reagent according to Singleton and Rossi, at λ = 760 nm with a Jenway 6105 UV/Vis [21]. TPC was calculated based on the calibration curve of 0, 6, 12, 18, 24 and 30 µg/mL gallic acid, generating the equation of y = 0.0187x − 0.0009, R^2^ = 0.9996. The results are expressed as mg gallic acid equivalent (Ga)/g dw.

### 2.4. Antioxidant Activity (FRAP)

The fruits’ antioxidant capacity was measured by the FRAP assay according to Benzie and Strain, at λ = 593 nm with a Jenway 6105 UV/Vis spectrophotometer [22]. FRAP was calculated based on the calibration curve of 0, 6, 12, 18, 24 and 30 µmol/L ascorbic acid, generating the equation of y = 0.0478x + 0.0104, R^2^ = 0.9997. The results are expressed as µmol ascorbic acid (As) equivalent/g dw.

### 2.5. Total Flavonoid Content (TFC)

Total flavonoid content was determined according to Sytar et al., aluminium chloride colorimetric method [23]. From the supernatant, 500 µL was added to 1.5 mL 95% EtOH, 0.1 mLA lCl_3_, 0.1 mL potassium acetate and 2.8 mL water. The absorbance was measured at λ = 415 nm using a Jenway 6105 UV/Vis spectrophotometer. TFC was calculated on the basis of the calibration curve of quercetin standard for which 0, 20, 40, 80, 120, 160 and 200 µg/mL quercetin was used, generating the equation of y = 0.0059x + 0.0361, R^2^ = 0.9989. Results are expressed as mg quercetin equivalent (Qe)/g dw.

### 2.6. Total Carotenoid Content (TC)

Total carotenoid content was measured a method described by Hornero-Mendez and Minguez-Mosquera [24]. Absorbance was measured by a Jenway 6105 UV/Vis spectrophotometer at λ = 452 nm and λ = 472 nm, characteristic absorption maximum of red and yellow carotenoids, respectively, results are expressed as mg/kg dw. The total carotenoid content was calculated using the following formula:

$$\mathrm{TC}\;(\text{µ}g/g)=\frac{A\times V_{\left(\mathrm{mL}\right)}\times 10^{4}}{A_{1\mathrm{cm}}^{1\%}\times W_{\left(g\right)}}$$

where A = absorbance (measured at either λ = 452 nm or 472 nm), $V_{\left(\mathrm{mL}\right)}$ = total extract volume, $W_{\left(g\right)\;}$ = sample weight and $A_{1\mathrm{cm}}^{1\%}$ = 2009 or 2144 (extinction coefficient of capsanthin and β-carotene in acetone, respectively). The sum of the two measurements gives the total carotenoid content.

### 2.7. Catalase Enzyme Activity (CAT)

The CAT activity measurement was carried out according to Xing et al. [25]. For the sample extract, sodium phosphate buffer (50 mM, pH 7) and 40 mM H_2_O_2_ as a substrate was measured. After the addition of the H_2_O_2_ the change in the absorbance was monitored at λ = 240 nm in 60 s intervals with a Jenway 6105 UV/Vis spectrophotometer. Results are displayed in U/g dw.

### 2.8. Peroxidase Enzyme Activity (POD)

The POD activity measurement was carried out according to Xing et al., samples were mixed with a buffer containing 8 mM guaiacol and 100 mM sodium phosphate pH 6.4. After the addition of 24 mM H_2_O_2_ as a substrate, the change in the absorbance was recorded at λ = 460 nm in 60 s intervals with a Jenway 6105 UV/Vis spectrophotometer. Results are displayed in U/g dw.

### 2.9. Superoxide Dismutase Enzyme Activity (SOD)

SOD activity was assayed by its ability to inhibit the photochemical reduction of nitroblue tetrazolium according to Beauchamp and Fridovich [26]. The reaction mixture contained 50 mM sodium phosphate buffer, 10 µM EDTA, 13 mM L-methionine, 75 µM nitroblue tetrazolium (NBT) and 2 µM riboflavin. During the reaction assay preparation, the mixture was kept in dark and to kickstart the reaction, the ready reaction mixture was illuminated with luminescent light for 10 min. Absorbance was measured at λ = 560 nm wavelength. Results are displayed in U/g dw.

### 2.10. Soluble Solid Content (SSC) and pH

The pH of the berries was measured with LAQUAtwin water quality pocket meter by Horiba (Kyoto, Japan), and their ºBRIX was determined by a digital refractometer, PR-201ᾳ, ATAGO^®^.

### 2.11. Determination of Colour Hue

Photos of the collected berries were taken in each phenophase upon sample collection with a Pentax GR2 camera. Average colour was determined with Adobe Photoshop CC (2019), the HEX values were converted to decimals in MS Excel version 2202 and were compared to the results of the other measurements.

### 2.12. RNA Isolation and Quantitative Real-Time PCR

Total RNA was isolated from the pericarp of fruits in each phenophase with Omega E.Z.N.A.^®^ Plant RNA Kit (Norcross, GA, USA). The integrity and quantity of the RNA samples were verified and measured by agarose gel electrophoresis and Nanodrop 1000 spectrophotometer by Thermo Fisher Scientific (Waltham, MA, USA), respectively. From the RNA cDNA was synthesized with RevertAid H Minus First Strand cDNA Synthesis Kit by Thermo Fisher Scientific (Waltham, MA, USA) with oligo-dT and random primers were applied, according to the manufacturer’s instructions. The qRT-PCR was carried out in a Stratagene MX3000p instrument using actin as reference gene. For PCRs we used Power Up™ SYBR™ Green Master Mix, Applied Biosystems by Thermo Fisher Scientific (Vilnius, Lithuania) according to the manufacturer’s instructions. Primers used in this study were either published previously by Aza-Gonzalez et al. or were designed by Primer3 using Zunla’s genome as a reference (Table 2) [4].

### 2.13. Statistical Analysis

The qRT-PCR data were evaluated via the ddCT method. The heatmap was constructed with TBtools using Eucledian distancing and cladogram branch type [27]. Microsoft Excel and IBM SPSS 25 were applied to calculate means, standard deviation of the means from the repeated measurements as well as Pearson correlation coefficient and F-value from analysis of variance (ANOVA).

## 3. Results and Discussion

The mutant breeding lines used for the study all shared the same genetic background with the ‘Soroksári’. The mutants were selected based on their anthocyanin accumulation patterns in their fruits or in their vegetative parts. Though they share the same genetic background they show large disparity in their nutritional and quality traits.

The pH of the berries in the GS1 stage ranged from 5.2 to 6.3 and in their biologically ripe stage from 5.2 to 5.8, hence no significant differences were observed between the colouration of the berries and their pH. Thus the berry colour is rather due to other genetic factors or other factors suggested by Láng [28]. Their soluble solid contents in the same phenophases ranged from 4.0 to 7.8 in GS1 and 4.5 to 8.6 ºBRIX in the biologically ripe stage. In accordance with Deepa’s suggestion, all data presented in this study are calculated on dry weight basis (dwb), but wherever is necessary data are also presented on fresh weight basis (fwb) [29].

### 3.1. Total Monomer Anthocyanins

**Figure 1:**
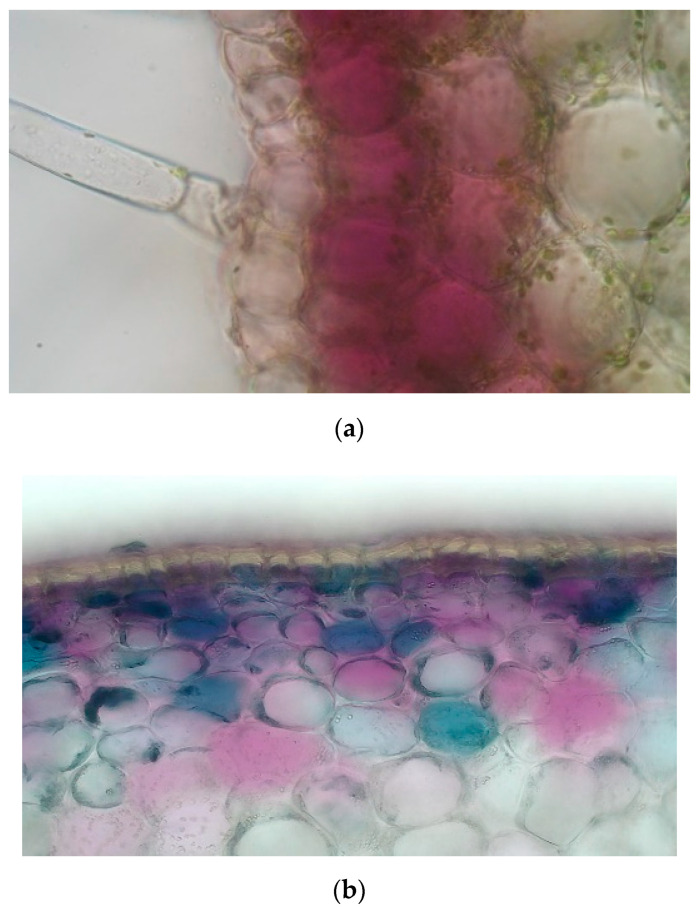
Cross-section of the hypocotyl (**a**) and berry (**b**) of ‘Pim. Ney.’.

To determine the presence of anthocyanins in the plant, microscopic pictures using Leica LEITZ DMRXE (Wetzlar, Germany) were taken. In addition to the fruits, from the ‘Pim. Ney.’, a photograph was also taken of its hypocotyl, since this extreme purple genotype accumulates anthocyanin in every phenophase in each organ. Cross-sections of both the hypocotyl and the fruit show that the anthocyanins are located in the vacuoles of the mesocarpic cells, and their intensity dilutes towards the inner mesocarp, corresponding to Lightbourn’s findings [5]. While in the hypocotyl only the first two layers of cells contain anthocyanins, the cross-section of the berry showed that under the cuticle there are five to six layers of mesocarp cells in the which are contained anthocyanis (Figure 1).

Thus far, the delphinidin-3-p-coumaroyl-runtinoside-5-glucoside is the main and only anthocyanidin found in the fruit, foliage and in the flower of pepper [30,31]. TMA was mostly recorded in the early phenophases except for ‘Pim. Ney.’ In two cases, anthocyanin was detected in the white-berried ones as well (11278 at breaker and in the ‘Soroksári’ at GS2 stage), although it could not be seen in the berries (Table 3). Sadilova et al. measured 321.5 µg cy-3-glu/g on fresh weight basis (fwb) in the peel of a *C. annuum* variety, whereas in the case of *C. annuum* we measured 15 times more (4866.47 µg cy-3-glu/g fwb equal to 56,575.27 µg cy-3-glu/g dw) and in the case of the *C. chinense* almost 130 times higher value on fwb (41,366.57 µg cy-3-glu/g fwb equal to 517,082.19 µg cy-3-glu/g dw) [31].

### 3.2. Total Polyphenolic Content

For the TPC measurement Folin–Ciocalteu assay was applied. The minor drawback of this method is that it also detects the additional capsaicinoids, ascorbic acid, flavonoids and minor phenolics, therefore generates higher values. As for the TPC values, generally in each genotype the lowest values were scored at the later stages, and usually higher values were observed in the economically ripe GS1 and GS2 stages on dry weight basis. However, when expressed on fwb an increasing trend was visible. The highest values were recorded for the ‘Pim. Ney.’, being significantly different from the other samples in every phenophase, and other studies also concluded that *C. chinense* varieties exhibit higher TPC values than *C. annuum* [32]. Higher values were expected at the GS1 stage in the lilac-berried mutants due to their elevated anthocyanin accumulation (43.11 to 43.73 mg/g); however, the white-berried genotypes at GS1 stage scored 1.5–2 times higher values (60.34 to 84.57 mg/g) (Table 3). Although there are studies indicating that there is no correlation in between maturity and TPC [33], when expressed on dry weight basis an overall decreasing trend can be seen during ripening, which supports Marín, Navarro, Ghasemnezh and Deepa’s studies [29,34,35,36]. When expressed on fwb, Chandel et al. measured from 0.621 to 1.690 mg/g, while we detected higher values, from 1.60 to 16.031 mg/g [37]. On the other hand, Howard et al., Sora et al. and Sim et al. found that TPC is greatly dependent on the sample material used and on the genotype studied [38,39,40].

### 3.3. Antioxidant Activity

Antioxidant activity of both fruits and vegetables is an important attribute when assessing their nutritional value and measuring it allows the determination of this without the measurement of each compound with antioxidant activity separately. FRAP assay was chosen to detect the antioxidant activity, which measures the antioxidant activity against the iron reducing capacity of the samples. This method is suitable for the analysis of the antioxidant capacity of water-soluble compounds, such as polyphenols, flavonoids, anthocyanins, ascorbic acid, etc. Most genotypes displayed high values of antioxidant capacity at GS1, followed by a decline at breaker stages, and a slight increase at biological ripeness. The highest antioxidant capacity was recorded in the ‘Pim. Ney.’, 1737.3 µmol/g dry weight at its breaker stage, although being the only pungent genotype, capsaicin could also add to the overall high values of AOX [41]. The lowest value was recorded at the breaker stage of the purple-berried mutant (11270), 108.40 µmol/g dry weight, which showed a 16-fold difference in between the studied genotypes at different phenophases (Table 3). AOX activity in addition to the GS1 stage differed significantly in each genotype, with the highest value recorded for the purple-berried ‘Pim. Ney.’ in each phenophase. Both the genotype and maturity affected the FRAP values significantly, e.g., in the case of ‘Pim. Ney.’ a significant increase was detected towards full ripeness, whereas a significant decrease was detected in 11274 when expressed on dry weight basis (Table 3).

### 3.4. Total Flavonoid Content

Flavonoids form an important group of health-promoting compounds since they exhibit free-radical scavenging activity, thus protecting the human body from oxidative stress. As ripening advances, the detected amount of flavonoids decreases, and this reduction may be due to the conversion to secondary metabolic phenolic compounds which is in agreement with the findings of Marín et al. and Ghasemnezhad et al. [34,36]. Interestingly, the extreme purple ‘Pim. Ney.’ did not score the highest values, whereas Ghasemnezhad detected the highest amount of flavonoids in a dark purple genotype both at ecological and biological ripeness [36]. Compared to Ana Karina et al., we detected 1.5- to 4.3-fold higher TFC in the red genotypes, 1.2 times higher in the orange genotype and half of the amount in the case of the yellow ripe berry [42]. When expressed on fresh weight, our results coincide with Garra et al., who reported TFC between 3.14 to 8.90 mg/g fresh weight, while we detected 0.18 to 7.89 mg/g on fwb [43].

### 3.5. Total Carotenoid Content

The ripeness of pepper is associated with the accumulation of carotenoids. A significant increase can be observed in the carotenoid content as ripening progresses (Table 3). The lowest amount was detected at the GS1 stage of 11270, 105.60 mg/kg, and the highest was scored at the ripe stage of cv. ‘Soroksári’, 6168.53 mg/kg. In this pepper an 8-fold increase was detected. Kilcrease et al. measured from 455.11 to 795.73 μg/g fwb in the pericarp of orange and red varieties, which is in line with our findings: the lowest TC content was 8.49 mg/kg fwb, the 11270 GS1 stage, whereas the highest, the ‘Soroksári’ fully ripe stage, was 697.28 mg/kg fwb [10]. When expressed on dwb, however, they measured from 1235.1 to 3049.1 mg/kg dwb in mature berries, whereas we detected from 717.20 in ‘Pim. Ney.’ to 6168.53 mg/kg dwb in the mature berries of the ‘Soroksári’ (Table 3) [11].

### 3.6. Enzymatic Activity

Studies are already available on the pattern of change of the non-enzymatic antioxidants, such as flavonoids, polyphenols, carotenoids, etc., throughout ripening of the pepper berry. However, there are only a few which deal with the enzymatic antioxidants over the course of maturation. Therefore, CAT, SOD and POD activity were monitored, as they serve as defence barriers against reactive oxygen species. The accumulation of these compounds is determined by several factors, both internal and external. External factors were minimized since the plants were kept under the same semi-controlled conditions, thus differences observed can be contributed to the genotype effect and ripening. The activity of CAT increased—though not significantly—in most of the studied genotypes, ‘Pim. Ney.’ being the only one where the increase was significant. In case of 11274 and 11280, however, a decrease from ecological to biological ripeness was observed, which is in line with the findings of Palma [44]. As for SOD activity, in the case of ‘Pim. Ney.’, 11263 and 11270, an increase was observed from GS1 to GS2, followed by a decrease at breaker stage, then an increase again at full maturity. Compared to GS1, there was an increase at the ripe stage of 11278, whereas in case of 11270, 11280 and ‘Soroksári’, a decrease was seen compared to GS1. Within genotypes, an overall increase was seen toward biological ripeness in the POD activity in the case of ‘Pim. Ney.’ and 11263–11278 breeding lines, whereas in the case of 11280 and ‘Soroksári’, a decrease was detected from GS1 to full ripeness (Table 3).

### 3.7. Correlation between Phytochemicals and AOX

As stated previously, ripening affects the phytochemical composition of the berries. To assess the degree of contribution of these compounds to the overall AOX of the berries, phytochemicals and antioxidant capacity as well as berry colour in each genotype in each phenophase were evaluated (Table 4). The FRAP and TPC values showed a strong positive correlation (r = 0.906), which is lower than that reported by Bogusz or Sora et al. but higher than that obtained by Deepa et al. (Table 4) [29,32,40]. TMA displayed strong positive correlation with both FRAP (r = 0.849) and TPC (r = 0.848), indicating that their presence is linked with the increased antioxidant capacity over the course of ripening. The correlation between carotenoids and FRAP was weaker (r = −0.150). This is due to the nature of the FRAP assay, since it requires acidic conditions (pH 3.6) within which carotenoids tend to undergo isomerization, thus losing their reducing activity [42].

In addition to assessing the contribution of the phytochemicals to the AOX, we also examined the effect of genotype and phenophase and their combination on both the AOX itself and on the related nutraceutical compounds as well. Guilherme et al. found that maturity affects the polyphenolic content and composition to a great extent while Howard et al. concluded that both the amount of phenolics and flavonoids are mostly affected by the cultivar [38,45]. Ghasemnezhad et al. also established the same conclusion, hence the changes in flavonoids depend on the cultivar rather than the maturity [36]. A two-way ANOVA was conducted and resulting F-values are summarized in Table 5. Numbers highlighted are the highest affecting factors in a group. Our results indicate that TMA, TPC and FRAP were affected by the genotype. On the contrary, the TFC was mainly influenced by the phenophase.

In the case of the TMA, an interaction between genotype and maturity could be demonstrated, F(18, 56) = 633.38 and *p* < 0.001. As for the TPC, F(18, 56) = 46.37 and *p* < 0.001. TFC was also affected significantly by both genotype and maturity, where F(18, 56) = 5255.05 and *p* < 0.001, as well as FRAP, where F(18, 56) = 9704.27 and *p* < 0.001 (Table 5). Although in most of the cases genotype was the most influencing factor, adjusted r squared in all four cases are between 0.978 and 1.00, meaning that variance in the phytonutrients is almost entirely attributable to the effect of genotype and maturity.

### 3.8. Regulation of Anthocyanin Biosynthesis

**Figure 2:**
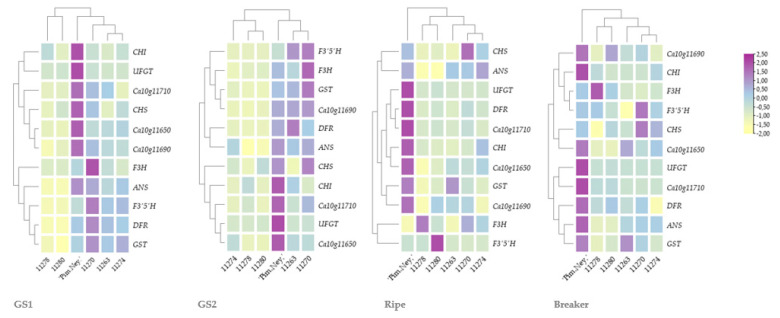
Fold expression pattern of the tested genotypes compared to cv. ‘Soroksári’ in 4 phenophases, pseudo-colour bar is showing the level of fold expression on a normalized scale.

The presence of anthocyanins positively correlated with the accumulation of transcripts of both regulatory and structural genes of the anthocyanin biosynthetic pathway (Figure 2, Table 6). Most studies conclude that R2R3-MYBs affect the expression of LBGs (F3′5′H, DFR, ANS, UFGT, GST); however, contradictory results are available on their effect on the EBGs (CHI, CHS, F3H) of the pathway [4,18,46]. The expression level of both ANS and DFR coincided with the higher expression of regulatory MYBs. Their expression was higher in those stages where the berries are still rich in anthocyanins; furthermore, a great fold of difference was observed in transcript levels between the anthocyanin-pigmented and anthocyanin-less genotypes (Figure 2). Interestingly, the transcript level of EBGs, CHS and F3H followed the expression of the studied MYB transcription factors, as opposed to Borovsky et al. and Aza-Gonzalez et al., who found that expression of CHS is comparable between the anthocyanin-rich and anthocyanin-less genotypes [4,18]. On the other hand, Stommel et al. detected higher transcript levels of CHS in the anthocyanin-pigmented genotypes; in fact, upon silencing the MYBa, Ochoa-Alejo et al. and Zhang et al. detected the down regulation of both LBGs and EBGs [46,47,48].

After comparing the expression level of the three R2R3-MYB transcription factors, it can be seen that the so-called MYBa (*Ca10g11650*) and the two other putative regulatory MYBs (*Ca10g11690* and *Ca10g11710*) were expressed at high levels in the lilac-berried genotypes, and their transcript level decreased in genotypes 11263, 11270 and 11274 as ripening advanced and the berries started to turn into their ripe colour. The extreme lilac genotype, ‘Pim. Ney.’, showed the highest log2 fold change in every phenophase. This might suggest that besides MYBa, these two other R2R3-MYBs, or a combination of them, regulate the anthocyanin synthesis in the berries of *Capsicums* (Table 6).

## 4. Conclusions

Taken together, significant changes both in between the genotypes and in between the different phenophases were observed in the case of enzymatic and non-enzymatic antioxidants. ‘Pim. Ney.’, the extreme purple genotype, exhibited outstanding results for TPC, TFC and FRAP in each phenophase compared to the rest of the samples. This might be due to the elevated amount of anthocyanins, which results in this rich dietary composition. On the other hand, the anthocyanin-pigmented *C. annuum* breeding lines did not live up to the expectations, since the white-berried breeding lines usually scored higher values for TPC, TFC and FRAP in the early phenophases. Based on this study, economically-ripe purple-berried breeding lines could not serve as functional food solely due to their anthocyanin build-up; however, other genotypes, such as the extreme purple ‘Pim. Ney.’, may be recommended as a dietary supplement or a partner in breeding programs for functional foods.

As for the regulation of anthocyanin biosynthesis, we found that besides *Ca10g11650*, two other putative regulatory MBYs (*Ca10g11690* and *Ca10g11710*) are also involved in the regulation of the pathway. However, to validate the exact function of the two putative regulatory MYBs, other approaches such as virus-induced gene silencing studies should be applied as well.

## Figures and Tables

**Table 1:** Codes, attributes and appearance of the breeding lines.

Name/Code	Description	Appearance
‘Pim. Ney.’	*C. chinense*, extreme purple throughout each phenophase	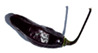
11263	matures from lilac to red, *pax*, *Leb*	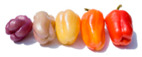
11270	matures from purple to yellow, *pax+*, *Leb*	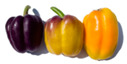
11274	matures from purple to red, *pax+*, *Leb-s*	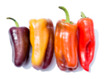
11278	matures from white to red, *pax*	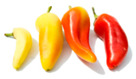
11280	matures from white to red, *pax+*, *Leb+*	
‘Soroksári’	matures from white to red, *asx*	

Note: *pax*, *pax+*—partially anthocyanin-less, *Leb*, *Leb+*, *Leb*-s—lilac economically ripe berry, *-s*: striped, *asx*—anthocyanin-less ‘Soroksári’ type.

**Table 2:** Primers used for qRT-PCR.

	Forward 5′–3′	Reverse 5′–3′	Source
*ACT*	GGACTCCGGTGATGGTGT	GTCCCTGACAATTTCTCGCTCAG	own design
*Ca10g11650*	TGGCTGCAGTTGGGATCTTT	TCCCAACCATCACTTTGTCCT	
*Ca10g11690*	TACTCGCCTTCTGAGGAAGGTA	TGGTACTTGAGAAGTTCCGAGG	
*Ca10g11710*	GACAGCGAGCGATGTGAAAA	GGCACTTGAGAAGTTCTGTGG	
*CHS*	AGGAGGTTCGAAGGGAACAA	CCATCACCAAAGAGTGCTTG	based on Aza-Gonzalez et al.
*CHI*	CCTCCTGGTTCTAACACCACC	CTTTGCGGCAGGTGAAACTC	
*F3H*	GGCATGTGTGGATATGGACC	CCTCCGGTGCTGGATTCTG	
*F3′5′H*	GATGGGGTGGCCGGTGATTG	GCCACCACAACGCGCTCG	
*DFR*	CTAACACAGGGAAGAGGCTGGTTT	AATCGCTCCAGCTGGTCTCATCAT	own design
*ANS*	ACCAGAACTAGCACTTGGCG	ACGCACTTTGCAGTTACCCA	
*UFGT*	GGATGGTGTCAAACAAGGC	GTTCAGTACAACACCATCTGC	based on Aza-Gonzalez et al.
*GST*	TGATTCTCTCGAGCAGAAAAAACC	TGGATAACCTTTGTTCATATATG	

**Table 3:** Means and standard deviation of the means of the enzymatic activity and phytochemicals measured in different phenophases (in dw).

GS1	TMA	TPC	FRAP	TFC	TC	CAT	SOD	POD
	µg cy-3-glu/g	mg Ga/g	µmol As/g	mg Qe/g	mg/kg	U/g	U/g	U/g
‘Pim. Ney.’	134,897.45 ± 723.37 a	116.78 ± 8.32 a	515.55 ± 6.26 a	45.38 ± 0.25 a	341.79 ± 113.45 a,b	8.97 ± 0.76 a,b	46.75 ± 2.53 a	67.90 ± 5.50 a,c
11263	10,222.68 ± 159.52 b	43.73 ± 0.12 b,c	281.67 ± 1.52 b	56.98 ± 0.2 b	273.21 ± 6.50 a,b	8.72 ± 1.12 a,b	42.53 ± 5.27 a	26.60 ± 13.55 b
11270	56,575.27 ± 1445.30 c	43.15 ± 0.13 b,c	359.28 ± 2.30 c	15.78 ± 0.04 c	105.60 ± 6.02 a	4.56 ± 0.68 a,b	64.84 ± 1.93 a	36.18 ± 2.44 a,b
11274	17,929.89 ± 6025.39 b	43.11 ± 0.26 b,c	366.58 ± 3.80 c	86.34 ± 0.54 d	448.47 ± 44.00 b	10.38 ± 2.14 a	58.48 ± 3.07 a	24.07 ± 2.10 b
11278	Nd ^1^	60.34 ± 3.64 b	232.84 ± 0.81 d	49.97 ± 0.30 e	489.41 ± 93.06 b,c	4.21 ± 0.83 a,b	48.85 ± 5.60 a	44.59 ± 4.79 a,b,c
11280	Nd ^1^	35.08 ± 0.32 c	455.04 ± 1.34 e	65.55 ± 0.16 f	432.05 ± 47.63 a,b	8.77 ± 1.45 a,b	158.46 ± 33.82 b	80,34 ± 6.75 c,d
‘Soroksári’	Nd ^1^	84.57 ± 0.56 d	511.03 ±1.76 a	17.01 ± 0.70 c	193.37 ± 55.77 a,b	3.18 ± 1.10 b	41.18 ± 3.01 a	115.92 ± 9.10 d
**GS2**								
‘Pim. Ney.’	461,480.11 ± 6274.54 a	107.13 ± 9.77 a	945.29 ± 1.33 a	50.54 ± 0.71 a	1108.41 ± 62.66 a	8.52 ± 1.55 a	163.79 ± 22.15 a	46.88 ± 0.08 a
11263	5615.80 ± 412.52 b	33.07 ± 1.02 b	181.02 ± 0.63 b	34.51 ± 0.03 b	473.84 ± 26.14 b	3.53 ± 0.23 b	55.76 ± 6.22 b	44.99 ± 1.42 a
11270	20543.74 ± 958.88c	25.57 ± 0.42 b	129.29 ± 0.83 c	22.62 ± 0.03 c	825.08 ± 58.10 a	5.14 ± 0.85 a, b	74.58 ± 1.51 b	62.75 ± 1.20 a
11274	7754.45 ± 630.72 b,c	44.75 ± 0.88 b,c	306.80 ± 1.46 d	83.23 ± 0.27 d	1493.24 ± 93.68 c	2.93 ± 0.13 b	28.15 ± 1.98 b	59.83 ± 0.78 a
11278	Nd ^1^	66.33 ± 5.30 c	358.24 ± 2.86 e	30.07 ± 0.07 e	451.59 ± 44.93 b	1.68 ± 0.12 b	29.37 ± 1.18 b	59.18 ± 5.97 a
11280	Nd ^1^	61.86 ± 7.26 c,d	411.35 ± 1.13 f	112.50 ± 0.17 f	1495.11 ± 49.65 c	2.32 ± 0.04 b	38.27 ± 0.40 b	145.09 ± 7.72 b
’Soroksári’	356.25 ± 22.85 b	42.74 ± 1.96 b,c	199.79 ± 0.88 g	10.48 ± 0.04 g	511.38 ± 41.75 b	4.27 ± 0.27 b	30.58 ± 7.84 b	68.89 ± 16.00 a
**Breaker**								
‘Pim. Ney.’	517,082.19 ± 13557.80 a	196.38 ± 2.19 a	1737.93 ± 8.96 a	43.93 ± 0.99 a	583.01 ± 42.03 a	15.60 ± 0.57 a	85.38 ± 9.31 a	129.80 ± 19.58 a
11263	Nd ^1^	21.72 ± 0.34 b	153.49 ± 0.27 b	9.39 ± 0.08 b	1085.18 ± 93.27 a,b	4.98 ± 0.72 b	37.09 ± 1.12 b	57.12 ± 0.79 b
11270	3962.81 ± 218.28 b	21.79 ± 0.89 b	108.40 ± 0.16 c	6.45 ± 0.06 c	1201.93 ± 32.17 b	5.45 ± 0.37 b	9.29 ± 4.36 c	64.02 ± 6.93 b
11274	2143.08 ± 318.16 b	24.42 ± 2.31 b,d	266.32 ± 0.33 d	6.29 ± 0.02 c	1129.88 ± 130.97 a,b	6.57 ± 4.74 a,b	14.50 ± 0.95 c	29.82 ± 0.97 b
11278	521.84 ± 60.26 b	87.66 ± 1.68 c	751.18 ± 1.33 e	11.97 ± 0.05 d	679.61 ± 167.51 a,b	3.84 ± 0.63 b	7.13 ± 0.34 c	30.91 ± 0.89 b
11280	Nd ^1^	30.80 ± 1.16 d,e	199.44 ± 0.38 f	7.15 ± 0.03 c	825.88 ± 73.16 a,b	6.08 ± 1.22 a,b	5.47 ± 0.72 c	33.12 ± 2.97 b
’Soroksári’	Nd ^1^	36.10 ± 2.04 e	275.31 ± 0.46 d	10.41 ± 0.07 b,d	1198.05 ± 121.63 b,c	7.17 ± 0.94 a,b	6.83 ± 0.11 c	55.32 ± 14.29 b
**Ripe**								
‘Pim. Ney.’	154,812.58 ± 77.25 a	98.08 ± 5.63 a	1155.14 ± 2.98 a	27.57 ± 0.27 a	717.20 ± 186.46 a	56.90 ± 2.94 a	115.58 ± 8.33 a	74.79 ± 10.59 a,b
11263	Nd ^1^	21.12 ± 0.59 b	245.63 ± 0.30 b	5.32 ± 0.02 b	1847.56 ± 366.00 a	8.85 ± 1.24 b	179.60 ± 29.65 a	165.10 ± 24.19 a,b
11270	352.08 ± 29.04 b	23.87 ± 0.49 b	217.02 ± 0.67 c	2.10 ± 0.05 c	1204.84 ± 189.71 a	5.07 ± 0.86 b	202.32 ± 155.68 a	198.94 ± 81.22 a
11274	Nd ^1^	21.83 ± 0.98 b	178.77 ± 0.55 d	10.22 ± 0.08 d	1651.30 ± 450.35 a	4.28 ± 0.23 b	16.48 ± 1.40 a	21.03 ± 6.23 b
11278	Nd ^1^	17.06 ± 0.75 b	133.71 ± 0.18 e	20.52 ± 0.08 e	2929.87 ± 510.87 a	9.91 ± 1.18 b	79.44 ± 19.74 a	75.59 ± 9.44 a,b
11280	Nd ^1^	42.94 ± 1.51 c	466.69 ± 0.63 f	10.55 ± 0.06 d	3874.30 ± 1065.33 a,b	3.41 ± 0.64 b	29.94 ± 15.11 a	25.15 ± 11.95 b,c
’Soroksári’	Nd ^1^	22.30 ± 0.84 b	207.80 ± 0.24 g	7.04 ± 0.05 f	6168.53 ± 921.44 b	7.94 ± 1.24 b	5.19 ± 2.07 a	6.18 ± 0.93 b,d

Note: Values in the same column and sub-table not sharing the same subscript are significantly different at *p* < 0.05 in the two-sided test of equality for column means. ^1^—This category is not used in comparisons because there are no other valid categories to compare; Nd stands for not detected.

**Table 4:** Pearson correlation coefficients between the enzymatic activity, phytochemicals and colour of pepper fruits.

	CAT	SOD	POD	FRAP	TPC	TMA	TFC	TC	Colour
CAT	1								
SOD	0.209	1							
POD	0.110	0.763 **	1						
FRAP	0.523 **	0.200	0.097	1					
TPC	0.322 **	0.079	−0.001	0.906 **	1				
TMA	0.272	0.301 *	0.226	0.849 **	0.848 *	1			
TFC	0.008	0.064	−0.052	0.208	0.281 **	0.218	1		
TC	−0.024	−0.025	−0.143	−0.150	−0.259 *	0.077	−0.194	1	
Colour	0.457 **	0.165	0.036	0.531 **	0.526 **	0.609 **	0.222 *	−0.242 *	1

*, ** Correlation is significant at the 0.05 level and 0.01 level, respectively.

**Table 5:** ANOVA F-value summary of 4 most important nutraceutical traits of 7 genotypes at 4 phenophases.

	TMA	TPC	TFC	FRAP
Genotype (G)	**5918.75**	**438.54**	12,311.20	**61,209.87**
Maturity (M)	537.56	86.37	**37,756.75**	4501.43
G x M	633.38	46.37	5255.05	9704.27

Note: values in bold are the highest affecting factors in a group.

**Table 6:** Log2 fold change of R2R3-MYBs compared to the cv. ‘Soroksári’ and extracts’ colouration throughout the four tested phenophases.

Gene ID	Phenophase	‘Pim. Ney.’	11263	11270	11274	11278	11280
*Ca10g11650*	GS1	1126.61	10.94	9.00	16.87	0.46	0.21
	GS2	2893.52	14.47	11.41	9.94	0.21	0.06
	Breaker	35.89	3.66	3.02	4.97	0.23	0.31
	Ripe	11.16	5.53	1.57	3.09	0.20	0.10
*Ca10g11690*	GS1	17.71	2.37	5.07	2.06	0.02	0.47
	GS2	16.35	18.11	23.61	0.20	0.03	0.28
	Breaker	5.25	1.47	1.53	0.18	0.06	0.44
	Ripe	3.36	0.53	0.86	0.05	0.03	0.24
*Ca10g1171*0	GS1	80.05	8.25	12.12	0.43	0.36	0.47
	GS2	302.22	3.54	29.17	0.50	1.23	0.24
	Breaker	177.36	0.46	1.74	0.32	0.85	0.48
	Ripe	171.32	0.36	0.29	0.36	0.67	0.42
Crude sample extracts’ colour	GS1→Ripe	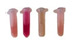	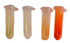	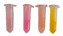	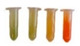	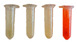	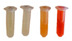

Note: Purple coloured cells indicate anthocyanin build-up in the berries.

## Data Availability

Data contained in the article are original.
